# Elevated homocysteine activates unfolded protein responses and causes aberrant trophoblast differentiation and mouse blastocyst development

**DOI:** 10.14814/phy2.15467

**Published:** 2022-09-18

**Authors:** Nadejda Capatina, Graham J. Burton, Hong Wa Yung

**Affiliations:** ^1^ Department of Physiology, Development and Neuroscience, Centre for Trophoblast Research University of Cambridge Cambridge UK; ^2^ Department of Clinical Neuroscience University of Cambridge Cambridge UK

**Keywords:** endoplasmic reticulum stress, hyperhomocysteinemia, placenta, pregnancy, trophoblast stem cells, unfolded protein response

## Abstract

Hyperhomocysteinemia may arise from folate/vitamin B_12_ deficiency, genetic polymorphisms, kidney disease, or hypothyroidism. It is associated with an increased risk of early pregnancy loss and placenta‐related complications of pregnancy, including pre‐eclampsia and fetal growth restriction. While the majority of studies of hyperhomocysteinemia focus on epigenetic changes secondary to metabolic disruption, the effects of homocysteine toxicity on placental development remain unexplored. Here, we investigated the influence of hyperhomocysteinemia on early blastocyst development and trophoblast differentiation. Exposure of cultured blastocysts to high homocysteine levels reduces cell number in the trophectoderm layer, most likely through increased apoptosis. Homocysteine also promotes differentiation of a trophoblast stem cell line. Both effects diminish the stem cell pool, and are mediated in an endoplasmic reticulum (ER) unfolded protein response (UPR^ER^)‐dependent manner. Targeted alleviation of UPR^ER^ may therefore provide a new therapeutic intervention to improve pregnancy outcome in women with hyperhomocysteinemia.

## Graphical abstract

• High concentration of homocysteine reduces cell number and activates endoplasmic reticulum unfolded protein response (UPRER) pathways exclusively in trophectoderm (TE) layer of mouse blastocyst.• Tauroursodeoxycholic acid (TUDCA), a bile acid, attenuates homocysteine‐mediated UPRER and abolishes apoptotic death in TE layer of blastocyst.• High homocysteine activates UPRER pathways in mouse trophoblast stem cells and promotes trophoblast differentiation.

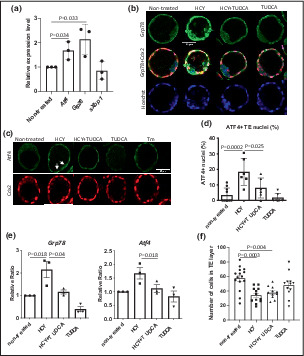

**Key points**

- High concentration of homocysteine reduces cell number in trophectoderm (TE) layer of mouse blastocyst.
- High homocysteine activates endoplasmic reticulum unfolded protein response (UPR^ER^) pathways exclusively in cells of TE layer.
- Tauroursodeoxycholic acid (TUDCA), a bile acid, attenuates homocysteine‐mediated UPR^ER^ and abolishes apoptotic death in TE layer of blastocyst.
- Homocysteine activates UPR^ER^ pathways in mouse trophoblast stem cells and promotes trophoblast differentiation.
- Our results suggest a potential therapeutic intervention to improve pregnancy outcome in women with hyperhomocysteinemia condition.

## 1 INTRODUCTION

Elevation of plasma homocysteine levels, a condition known as hyperhomocysteinemia, can arise from folate and/or vitamin B_12_ deficiency, genetic mutation of key enzymes involved in one‐carbon metabolism, and/or other health conditions including kidney disease and hypothyroidism (Catargi et al., 1999; Ducker & Rabinowitz, 2017; Friedman et al., 2001; Goyette et al., 1994). This medical condition affects ~21% of women of childbearing age in the UK (Sukumar et al., 2016). There is mounting epidemiological evidence that hyperhomocysteinemia is associated with an elevated risk of early pregnancy loss and recurrent miscarriage, as well as placenta‐related complications of later pregnancy, including fetal growth restriction and early‐onset pre‐eclampsia (Chaudhry et al., 2019; Hague, 2003; Mascarenhas et al., 2014; Ray & Laskin, 1999). A strong association exists between maternal plasma homocysteine concentrations during the first trimester and early pregnancy loss, with a mean concentration of 24.7 ± 4.5 μmol/L in cases of miscarriage compared to 13.5 ± 7.5 μmol/L in ongoing pregnancies (*p* = 0.0002) (Mascarenhas et al., 2014). Another cohort study involving 8085 women demonstrated that hyperhomocysteinemia increases the risk of placenta‐related complications over twofold (Chaudhry et al., 2019). In both studies, serum folate levels and folic acid supplementation were not associated with pregnancy outcome (Chaudhry et al., 2019; Mascarenhas et al., 2014), eliminating hypomethylation and thereby epigenetic influences linked to folate deficiency as a cause (Crider et al., 2012). Rather, the data suggest a direct toxic effect of hyperhomocysteinemia on development of the placenta, which in turn would interfere with fetal development (Perez‐Garcia et al., 2018).

Homocysteine (HCY) is a non‐proteinogenic amino acid that is highly reactive and can modify intracellular proteins, altering their signaling capabilities (Gurda et al., 2015). For instance, hyperhomocysteinemia results in formation of homocysteine‐thiolactone, a reactive thioester that can modify lysine residues through N‐homocysteinylation and leads to protein misfolding (Jakubowski, 1999; Sharma et al., 2014). Aggregation of misfolded proteins induces endoplasmic reticulum (ER) stress and activates the unfolded protein response (UPR^ER^; Ron & Walter, 2007). Indeed, high levels of HCY activate UPR^ER^ signaling pathways in vitro and in vivo (Hosoi et al., 2010; Martinez‐Pizarro et al., 2016; Reddy et al., 2019; Roybal et al., 2004). Both homocysteinylation and UPR activation are linked to the pathophysiology of specific human disorders, including cardiovascular disease, Alzheimer's disease, diabetes, renal disease, and reproductive problems (Forges et al., 2007; Jakubowski, 1999; Wang & Kaufman, 2012). Furthermore, protein aggregation disrupts placental development and trophoblast differentiation in mice (Watson et al., 2007, 2011).

The primary aim of the ER unfolded protein response (UPR^ER^) is to restore normal cellular function, but if this fails apoptosis may be induced to eliminate severely damaged cells and preserve tissue integrity (Ron & Walter, 2007). The UPR^ER^ signaling pathway consists of three major axes: the Perk/eIF2α/Atf4 pathway, the Ire1/Xbp1 pathway, and the Atf6 pathway. Each pathway is activated through a unique sensor protein residing on the ER membrane that initiates several downstream effectors (Ron & Walter, 2007). In addition to its homeostatic functions, increasing evidence supports a role for the UPR^ER^ signaling pathways in regulation of embryonic stem cell differentiation (Berger et al., 2016; Garcia‐Prat et al., 2017; Heijmans et al., 2013; Kratochvilova et al., 2016). For example, induction of ER stress in intestinal organoid cultures leads to a complete loss of the intestinal epithelial stem cell markers *Lgr5* and *Olfm4*, while inhibition of eIF2α phosphorylation restores the stem cell state (Heijmans et al., 2013). Indeed, our recent study demonstrates that ER stress modulates differentiation of stem cell populations in the extraembryonic tissues that give rise to the placenta (Capatina et al., 2021) and may contribute to placental dysmorphogenesis (Yung et al., 2012).

The trophectoderm (TE) layer of the blastocyst differentiates into separate trophoblast cell lineages (Artus & Hadjantonakis, 2012; Rossant & Tam, 2009) that play different, but essential, roles at the maternal‐fetal interface (Watson & Cross, 2005). In the mouse, these include the extraembryonic ectoderm that gives rise to multinucleated syncytiotrophoblast that together with fetal capillaries forms the labyrinth layer where maternal‐fetal exchange takes place. The extraembryonic ectoderm also gives rise to the ectoplacental cone from which several trophoblast giant cell (TGC) subtypes, and the spongiotrophoblast cells and glycogen trophoblast cells of the endocrine junctional zone of the placenta differentiate. For the placenta to form, there must be a controlled balance between trophoblast stem cell (TSC) self‐renewal and differentiation. These processes are normally regulated by growth factors acting through a complex network of transcription factors (Latos & Hemberger, 2016) including Cdx2, Esrrb and Eomes that are crucial for TSC survival and self‐renewal (Russ et al., 2000; Strumpf et al., 2005). Depletion of the TSC pool through apoptosis or premature differentiation will have a critical impact on placental development.

The mechanisms by which hyperhomocysteinemia mediates early pregnancy loss are currently unclear. We recently demonstrated that activation of ER stress pathway reduces cell proliferation in the TE layer of mouse blastocysts and promotes premature differentiation of TSCs, resulting in early pregnancy loss that can be partially reversed by tauroursodeoxycholic acid (TUDCA), an ambiphilic bile acid (Capatina et al., 2021). TUDCA is a chemical chaperone and has been demonstrated to alleviate ER stress in a mouse model of type 2 diabetes (Capatina et al., 2021; Ozcan et al., 2006). In this study, we first use an ex vivo blastocyst culture system to expose blastocysts to high concentrations of HCY, mimicking hyperhomocysteinemia, and examine whether HCY influences development of blastocysts and if this is mediated by UPR^ER^ pathways. We next use mouse TSCs in in vitro culture to examine whether high HCY modulates TSC differentiation via UPR^ER^‐dependent pathways. Our aim is to elucidate a molecular mechanistic linkage between hyperhomocysteinemia and potential poor placental development.

## 2 MATERIALS AND METHODS

### 2.1 Mice

All animal work was performed under the Animals (Scientific Procedures) Act 1986 Amendment Regulations 2012 following ethical review by the University of Cambridge Animal Welfare and Ethical Review Body (AWERB). All investigators understood and worked by the ethical principles and standards discussed by Grundy (2015). Mice were housed in M3 conventional cages (NKP, UK), at 55% humidity and 21°C, with a 12 h light cycle. Mice were fed RM3(E) diet (Special Diet Services) ad libitum from weaning. C57Bl/6 mice were purchased from Charles River Laboratories and bred in‐house. Pregnant females were identified on the basis of a vaginal plug. Noon of the day the vaginal plug was identified was dated as E0.5.

### 2.2 Embryo and placenta dissection and phenotyping

Pre‐implantation embryos were collected at E2.5 and E3.5 by flushing the oviduct and uterus with M2 medium (Sigma, M7167) as previously described (Piliszek et al., 2011). The embryos were either processed for immunostaining or cultured for 24 or 48 h in EmbryoMax® KSOM medium (MR‐020P, Sigma) in a humidified incubator at 37ᴼC with 5% CO_2_. All medium drops were covered with Oil for Embryo Culture (9305, Irvine Scientific) during embryo manipulation or culture.

### 2.3 Homocysteine (Hcy) treatment of blastocysts

Morulas were collected at E2.5 and cultured in 100 μM DL‐homocysteine (Sigma, H4628) in EmbryoMax® KSOM medium in the presence or absence of 500 μM sodium tauroursodeoxycholate (TUDCA; T0266, Sigma) for 48 h. Blastocysts treated with 2.5 μg/ml tunicamycin (Tm, Sigma, T7765) for 2 h were used as positive controls. Both TUDCA and DL‐homocysteine were prepared and dissolved in ultra‐pure milli‐Q water.

### 2.4 Trophoblast stem cells

The TSC EGFP‐TS line have been described previously (Tanaka et al., 1998). TSCs were acquired at passage 28 (P28) and were successfully maintained in culture up to P70. TSCs were cultured as previously described in humidified incubator at 37°C with 5% CO_2._ Cells were grown for in TS complete medium (30% TS base medium, 70% MEF‐conditioned medium (MEF‐CM), 25 ng/ml Fgf4 (Peprotech, 100–31), 1 μg/ml heparin (Sigma, H3393)). TS base medium contained RPMI 1640 with L‐Glutamine (Invitrogen, 21875‐034), 20% fetal bovine serum (Invitrogen, 10270106), 1 mM sodium pyruvate (Invitrogen, 11360039), 50 μM 2‐mercaptoethanol (Invitrogen, 31350‐010), and 50 U/ml Pen+Strep (Invitrogen, 15140‐122). MEF‐CM medium was prepared by culturing irradiated MEF cells in TS base medium for 48 h.

Prior to experimental treatments, TSCs were plated at a density of 70,000 cells/ml in 3 mm culture dishes (SIAL0165, Sigma). For experimental treatments, TS complete medium was removed from TSCs after 48 h culture and new TS complete medium only was added to vehicle‐control, or containing HCY. Cells cultured in TS base medium were included as positive control.

### 2.5 Immunofluorescence of TSCs and blastocysts

TSCs were washed in phosphate buffered saline (PBS) (Oxoid, BR0014G) and fixed in 4% paraformaldehyde (BDH, 294474 L) in PBS at room temperature (RT) for 20 min. Permeabilization, washes and antibody incubations were performed using buffer containing 0.1% Saponin (Sigma, S‐2149) and 1% Bovine Serum Albumin (BSA) (Sigma, A3059) in PBS. Blastocysts were fixed in 4% PFA and incubated in permeabilization solution containing 0.25% Triton X‐100 and 0.1% BSA. TSCs and blastocysts were incubated with primary antibodies diluted in 0.01% Tween‐20, 1% BSA overnight at 4°C. The following antibodies and dilutions were used: 1:150 anti‐GRP78 (Abcam, ab21685), 1:200 anti‐CDX2 (Biogenex, MU392‐UC), 1:100 anti‐ATF4 (Cell Signaling Technology, D4B8), 1:100 anti‐FoxO3A (Cell Signaling Technology). After washes in PBS, TSCs and blastocysts were incubated in 1:400 of Alexa‐Fluor conjugated secondary antibodies (488 and 568) (Thermofisher scientific) for 1 h incubation at RT in the dark. Next, TSCs and blastocysts were washed three times for 5 min each with the last wash containing 1 μg/ml Hoechst Dye 33342 (Sigma, B2261). TSCs were mounted using Vectashield antifade medium (Vector, H‐1200) onto SuperFrost® Plus microscope slides (ThermoFisher scientific) and imaged using a Zeiss AxioImagerA1 and AxioVs40 v.4.8.2.0 software. Blastocysts were assembled into individual drops in Ibidi glass‐bottom μ‐dishes (81158, Ibidi) and imaged using a Leica SP8 Advanced Confocal microscope. TE and ICM cell numbers were counted separately using plug‐in Fiji in Image J.

### 2.6 RNA isolation, reverse transcription and PCR or qPCR

For placentas at E9.5, total RNA was extracted using either an RNeasy Mini Kit (Qiagen, 74104) for TSCs or RNeasy Plus Universal Mini Kit (Qiagen, 73404) following the manufacturer's instructions. The concentration of RNA was determined using a NanoDropTM spectrophotometer (Thermo Scientific). The ratio of absorbance between 260 nm/280 nm for all samples was over 2. RNA integrity was analyzed by agarose gel electrophoresis and only samples showing stronger band intensity in 28S than 18S were used for reverse transcription. The reverse transcription was performed using 1 μg total RNA with SuperScript® III Reverse Transcriptase (ThermoFisher, 18080044) as previously described (Yung et al., 2019). The qPCR was performed according to the MIQE guidelines (Bustin et al., 2009). The reverse transcription reaction was diluted 1:5 for subsequent qPCR using SYBR® Green JumpStart™ Taq ReadyMix™ (Sigma, S4438) or Absolute qPCR ROX Mix (Thermo Scientific, AB‐1138/B) on the MJ Research DNA Engine Opticon 2 continuous fluorescence detector and Opticon Monitor v3.1.32 software. To reduce variation from pipetting, 3 μl of diluted cDNA was added to the reaction mixture and reactions were performed in technical triplicates. Primers for SYBR® Green qPCR were synthesized by Sigma (Table S1). The gene expression levels were calculated using the threshold cycle method (2‐ΔΔCT method). Data were normalized to the geometric mean of *Hprt* and *Sdha* internal controls as described in (Vandesompele et al., 2002).

For cultured blastocysts, a pool of 4–8 embryos from the same treatment were lysed with 100 μl RTL buffer and RNA extraction was performed using the RNeasy Micro Kit (74004, Qiagen) according to the manufacturer's instructions. Reverse transcription was performed using the Sensiscript® RT Kit (205211, Qiagen). qPCR was performed as described above.

### 2.7 Statistical analysis

All statistical analyses were performed using statistical package GraphPad Prism 9. Details of statistical analysis for each study are included in the respective figure legend. All *N*‐values in figure legend were number of independent biological replicates. All tests were two‐sided and *p* < 0.05 was defined as statistically significant. Datasets were tested for normality using the D'Agostino‐Pearson omnibus test and Shapiro–Wilk test. Non‐parametric tests were used for datasets that were not normally distributed.

## 3 RESULTS

### 3.1 Homocysteine reduces TE cell number and promotes apoptosis in blastocysts

**FIGURE 1:**
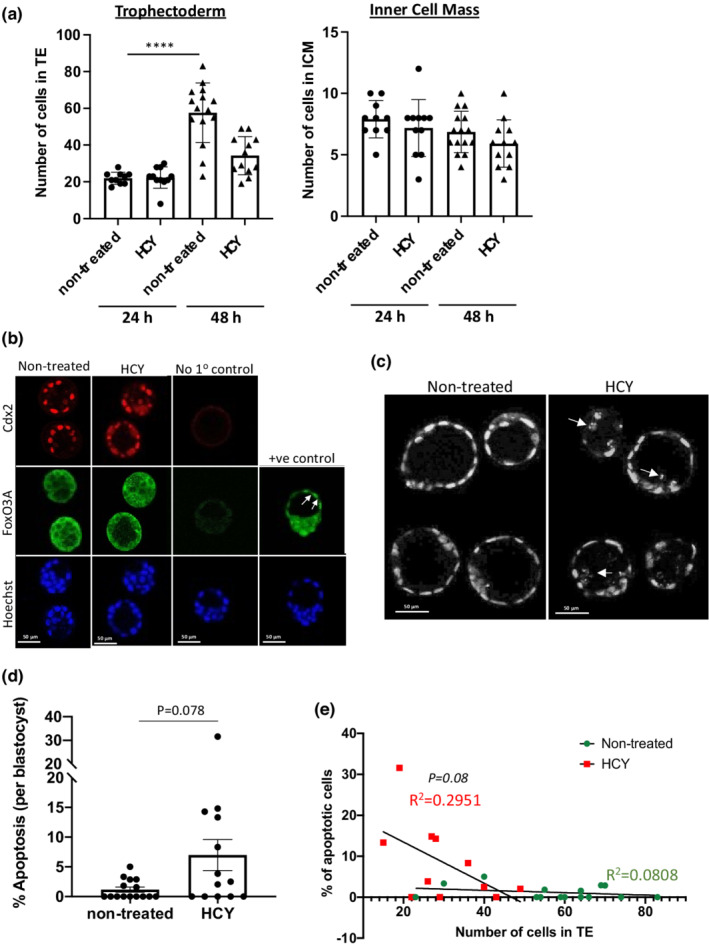
Blastocysts treated with HCY exhibit low cell number in trophectoderm and is associated with higher apoptotic incidence. Blastocysts were harvested at E2.5 from C57/Bl6 mouse before subjecting to ex vivo culturing for 24 or 48 h and either fixation for immunostaining or pooling for RNA isolation for RT‐qPCR analysis. (a) Total number of cells in trophectoderm (TE) layer is reduced in HCY‐treated blastocysts but not in inner cell mass (ICM) after 48 h. Blastocysts were immunostained for trophoblast stem cell markers Cdx2 and counterstained for Hoechst 33342. Cdx2‐positive cells were in TE, while Cdx2 negative cells were cells in ICM. The numbers of Cdx2+ and Cdx2− cells were counted separately after 24 and 48 h incubation. Data are mean ± SD, *n* = 10–15 blastocysts each group. One‐way ANOVA with Tukey's multiple comparison test. (b) Immunostaining for Cdx2 (red) and FoXO3A (green) and counterstained with Hoechst 33342 for nuclei. Blastocysts treated with Tm were used as positive control for FoXO3A nuclear translocation. All images were taken using same settings with Leica SP8 confocal microscope. For “No 1o control”, primary antibody was not added to the cells. Scale bar = 50 μm. (c, d) HCY induces apoptosis in cells in blastocysts. (c) Hoechst 33342 staining showed fragmented nuclei present in HCY‐treated blastocysts. (d) Number of fragmented nuclei was counted and normalised with total number of cells. Scale bar = 50 μm. Data are presented as mean ± SD, *n* = 13–15 each group and is expressed as percentage of apoptotic cells per blastocyst. Non‐parametric Mann Whitney test. (e) A negative correlation between percentage of apoptotic cells and number of cells in TE layer. Scatter plot was constructed between % of apoptotic cells and number of cells in TE and a linear regression line was fitted. Two‐tailed Pearson *r* test was performed.

We first investigated the effects of a high HCY concentration on blastocyst development using an ex vivo model. Normal physiological plasma homocysteine concentrations range between 5 and 15 μM in humans, while the concentration may exceed 100 μM in severe cases of hyperhomocysteinemia (Kang et al., 1992). To elucidate whether high HCY levels affect pre‐implantation embryo development, morulas were harvested from C57Bl/6 mice at embryonic day 2.5 and cultured in normal medium or medium supplemented with HCY (100 μM) for 48 h. Morulas cultured under both conditions formed blastocoels and progressed to blastocysts. The numbers of TE and inner cell mass (ICM) cells were counted separately after 24 and 48 h of culture. The threefold increase (*p* < 0.0001) in TE cells observed in the control blastocysts between 24 and 48 h of culture was not observed in HCY‐treated blastocysts (Figure 1a), suggesting that the high HCY concentration either suppressed cell proliferation or increased apoptosis. By contrast, there was no effect of HCY treatment on ICM cell number after 24 or 48 h (Figure 1a).

Nuclear translocation of the FoxO transcription factor family, including FoxO3A, has been implicated in cell‐cycle arrest, inhibiting cell proliferation (Medema et al., 2000) and causing developmental delay in pre‐implantation embryos in response to stress (Kuscu & Celik‐Ozenci, 2015). However, HCY‐treated blastocysts showed similar protein level and cytoplasmic localization of FoxO3A to the non‐treated controls, while there was clear nuclear localization of FoXO3A in positive controls treated with tunicamycin (Tm) (Figure 1b). These data suggest that cell‐cycle arrest was unlikely to cause the reduced TE cell number following HCY treatment.

On the other hand, application of 100 μM HCY for 48 h caused a trend toward increased (*p* = 0.078) apoptosis in the TE layer as indicated by nuclear fragmentation (Figure 1c,d). A negative correlation was found between the percentage of apoptotic cells and the number of TE cells in HCY‐treated blastocysts (*R* ^2^ = 0.2951, *p* = 0.08, two‐tailed Pearson r test), but not in non‐treated control blastocysts (*R* ^2^ = 0.0808, *p* = 0.31) (Figure 1e). These data suggest that increased apoptosis is likely a major contributor to the reduced TE cell number in HCY‐treated blastocysts, although they failed to reach statistical significance.

### 3.2 High homocysteine levels induce ER stress in TE but not ICM cells

**FIGURE 2:**
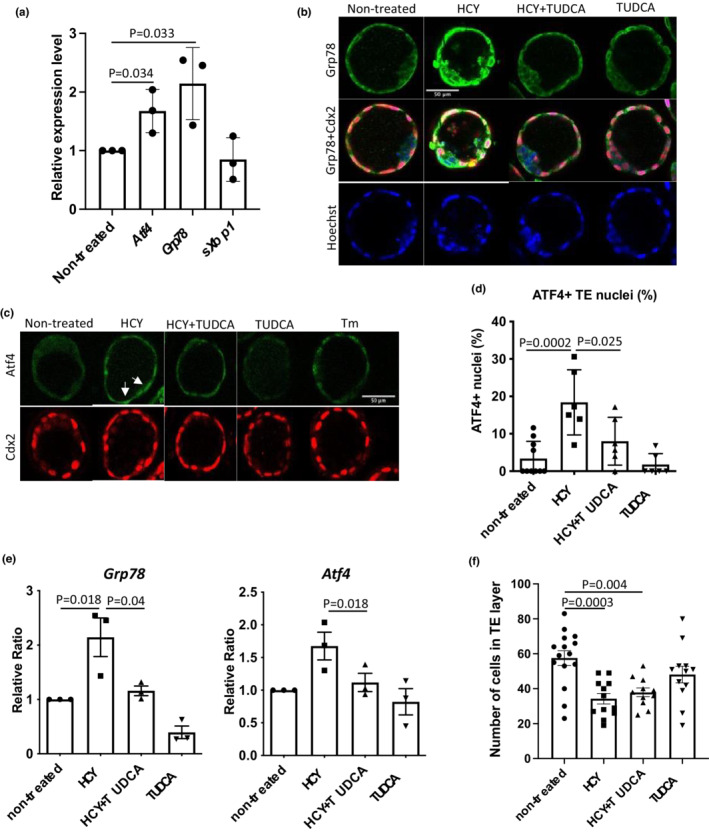
HCY activates UPR^ER^ pathways in blastocysts ex vivo and is abolished by TUDCA. A total of 74 morulas was harvested from 11 C57/Bl6 females at 2.5 dpc and cultured in KSOM medium in the presence or absence of HCY (100 μM) or TUDCA (500 μM) or a combination of both for 48 h. Blastocysts were either fixed for immunostaining or total RNA was collected from a pool of 4–8 blastocysts per group for RT‐qPCR. (a) Elevation of *Atf4* and *Grp78* transcripts in HCY‐treated blastocysts. (b–d) ER stress is presented exclusively in TE layer of the HCY‐treated blastocysts. Double immunostaining was performed with (b) Grp78 and Cdx2 (for TE lineage) or (c) Atf4 and Cdx2 antibodies. All images were taken using same settings with a Leica SP8 confocal microscope. Scale bar = 50 μm. (d) Number of Atf4‐positive nuclei in trophectoderm layer was counted and presented as percentage of total number of nuclei in trophectoderm layer per embryo. Data are presented as mean + SD, *n* = 6–9 embryos. One‐way ANOVA with Tukey's multiple comparison test. (e) TUDCA abolishes HCY‐mediated elevation of *Grp78* and *Atf4* transcripts, indicating alleviation of ER stress. Results were quantified and presented as ratio relative to untreated controls which was set as 1. Data are presented as mean+SD, *n* = 3 independent experiments. One‐way ANOVA with Bonferroni's multiple comparisons test. (f) TUDCA does not restore cell number in HCY‐treated blastocysts. Total numbers of cells in trophectoderm (TE) layer and inner cell mass (ICM) were counted separately. Data are mean ± SD, *n* = 11–15 blastocysts each group. One‐way ANOVA with Bonferroni's multiple comparisons test.

High HCY leads to activation of UPR^ER^ signaling pathways in vitro and in vivo (Hosoi et al., 2010; Martinez‐Pizarro et al., 2016; Reddy et al., 2019; Roybal et al., 2004). Therefore, we examined whether high concentrations of HCY cause ER stress and activate UPR^ER^ signaling pathways in mouse blastocysts. Total RNA was isolated from a pool of 4–8 blastocysts cultured under non‐treated control and HCY‐treated conditions and used to measure expression of transcripts in all three arm of UPR^ER^ signaling pathways, including *Atf4* (Perk axis), *Grp78* (Atf6/Ire1 axes), and spliced variant of *Xbp1* (*sXbp1*)(Ire1 axis) by RT‐qPCR. Compared to non‐treated controls, HCY‐treated blastocysts showed a 1.6‐ (*p* = 0.034) and 2.1‐fold (*p* = 0.033) increase in expression of *Atf4* and *Grp78*, respectively, while there was no change in *sXbp1* transcripts (Figure 2a). Immunostaining for Grp78 and Atf4 proteins showed that ER stress was induced primarily within the TE layer of HCY‐treated blastocysts (Figure 2b,c), consistent with our recent study showing that cells in the TE layer are more susceptible to ER stress (Capatina et al., 2021). Furthermore, there was a significant increase (*p* = 0.0002) of nuclear Atf4 in TE cells of HCY‐treated blastocysts (Figure 2d), indicating potential functional activity of the Atf4 transcription factor (Lange et al., 2008). Blastocysts exposed to Tm were used as a positive control and showed nuclear localization of Atf4 (Figure 2c).

To test whether the apoptosis observed above is mediated through ER stress pathways, we co‐treated blastocysts with HCY and the chemical chaperone TUDCA, which alleviates ER stress in placenta in vivo (Capatina et al., 2021). Co‐application of TUDCA led to a significant reduction of ER stress induced by high HCY levels as indicated by significant decreases in *Grp78* (*p* = 0.04) and *Atf4* (*p* = 0.035) mRNA expression (Figure 2e) and Atf4 protein nuclear localization (*p* = 0.025) compared to blastocysts treated with HCY alone (Figure 2c,d). Despite the reduction of ER stress, there was no significant reduction in the average percentage of apoptotic cells in HCY‐treated blastocysts (Figure S1). However, we did observe that fewer blastocysts exhibited apoptotic cells in the TE layer in blastocysts exposed to HCY + TUDCA (4 out of 12) compared to HCY alone (8 out of 13) (Figure S1). TUDCA treatment also reduced the incidence of apoptosis in the control blastocysts (Figure S1, non‐treated vs TUDCA alone). Furthermore, co‐treatment with HCY and TUDCA had little impact on restoring average TE cell number in blastocysts (Figure 2f). However, frequency distribution analysis indicates that following exposure to TUDCA, TE cell numbers were partially restored in individual blastocysts. In blastocysts containing fewer than 30 cells in the TE layer there was a 50% reduction in TE cell number in 6 out 12 (50%) treated with HCY alone compared to only 3 out 11 (27%) treated with HCY + TUDCA (Figure S1). Nevertheless, these results indicate the involvement of other mechanisms in the regulation of cell proliferation in the TE layer that may act independent of ER stress pathways and apoptosis.

### 3.3 Homocysteine activates UPR^ER^ signaling pathways in TSCs

Our recent publication demonstrates ER stress can modulate differentiation of mouse TSCs (Capatina et al., 2021). Therefore, we next investigated whether HCY can also modulate TSCs differentiation via UPR^ER^ signaling pathways. We used an established primary TSC line (Tanaka et al., 1998) to investigate whether high HCY induces ER stress in TSCs. Experiments were performed in the standard medium commonly used to maintain TSCs in their self‐renewing stem cell state that contains fetal bovine serum (FBS), FGF4, heparin, and mouse embryonic fibroblast‐conditioned medium (MEF‐CM), referred to here as “complete” medium (Tanaka et al., 1998). In contrast, removal of FGF4, heparin, and MEF‐CM from the culture facilitates TSC differentiation into different trophoblast lineages and was used as a positive control (Erlebacher et al., 2004; Tanaka et al., 1998). This is referred to as ‘TS base’ medium.

**FIGURE 3:**
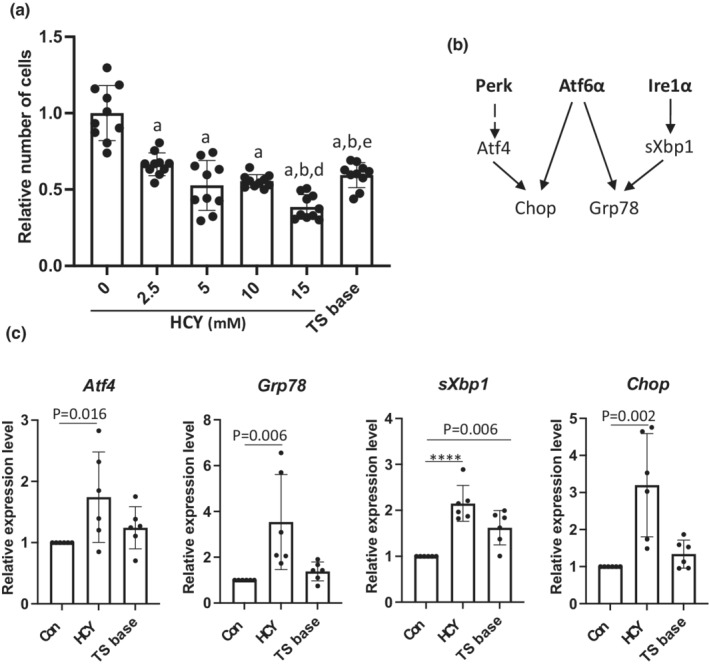
Homocysteine diminishes trophoblast stem cell population in a dose‐dependent manner and activates Perk and Ire1 axes of UPR^ER^ signalling pathways in mouse trophoblast stem cells (TSCs). TSCs were cultured in TS base medium or in complete medium and increasing concentrations of HCY (from 2.5 to 15 mM) or TS base medium for 48 h. (a) Cells were fixed for nuclear staining of Hoechst 33342. The numbers of nuclei were counted and was expressed as relative number of cells. Results were from 10 fields from 2 independent experiments. Data are presented as mean ± SD. One‐way ANOVA with Tukey's multiple comparisons test in comparison to untreated control (0). “a”, “b”, “c”, “d” and “e” indicates *p* < 0.05 compared to 0, 2.5, 5, 10, and 15 mM respectively. (b) A schematic diagram showing the three axes of UPR^ER^ signalling pathways and their downstream effectors. (c) Total RNA was isolated after 48 h for RT‐qPCR analysis of ER stress marker expression. Data are presented as relative ratio to untreated control (Con) (mean ± SD), *n* = 6 biological replicates per group. RM one‐way ANOVA with Dunnett's multiple comparisons test was performed in comparison to Con. ****p < 0.0001.

The 100 μM concentration of HCY used in the blastocyst experiments did not provoke any response in TSCs. Therefore, we performed a dose–response study in TSCs using higher concentrations of HCY from 2.5 to 15 mM for 48 h. Although homocysteine had little impact on cell death in TSCs at concentrations up to 15 mM (2.1%, *p* < 0.0001; Figure S2a,b), the increased levels gradually reduced the number of TSCs by 33% (*p* < 0.0001) and 61% (*p* < 0.0001) at 2.5 and 15 mM, respectively (Figure 3a). These data suggest that sublethal concentrations of HCY reduce TSC proliferation, consistent with the blastocyst results above. Total RNA was collected for analysis of transcript levels of genes involved in UPR^ER^ signaling pathways, including *Atf4*; *Chop* (both Perk and Atf6 axes); *Atf6α* (Atf6 axis); *sXbp1* and *Grp78* by RT‐qPCR (Figure 3b). Treatment with high HCY concentrations induced a dose‐dependent increase of all transcripts except *Atf6α* (Figure S3a). HCY at 10 mM was chosen for subsequent studies because its sublethal dosage had less impact on cell number compared to 15 mM. At 10 mM, HCY significantly increased transcript levels of *Atf4* by 0.74 fold (*p* = 0.016), *Chop* by 3.20 fold (*p* = 0.002), *Grp78* by 3.54 fold (*p* = 0.006) and *sXbp1* by 2.15 fold (*p* < 0.0001) while *Atf6α* showed no change (Figure 3c; Figure S3b). In comparison, incubation in TS base medium caused moderate elevation of *sXbp1* and *Atf6α* by 0.62 fold (*p* = 0.006) and 0.82 fold (*p* = 0.003), respectively, (Figure 3c; Figure S3b), consistent with our previous report (Capatina et al., 2021).

### 3.4 Homocysteine promotes differentiation of TSCs

**FIGURE 4:**
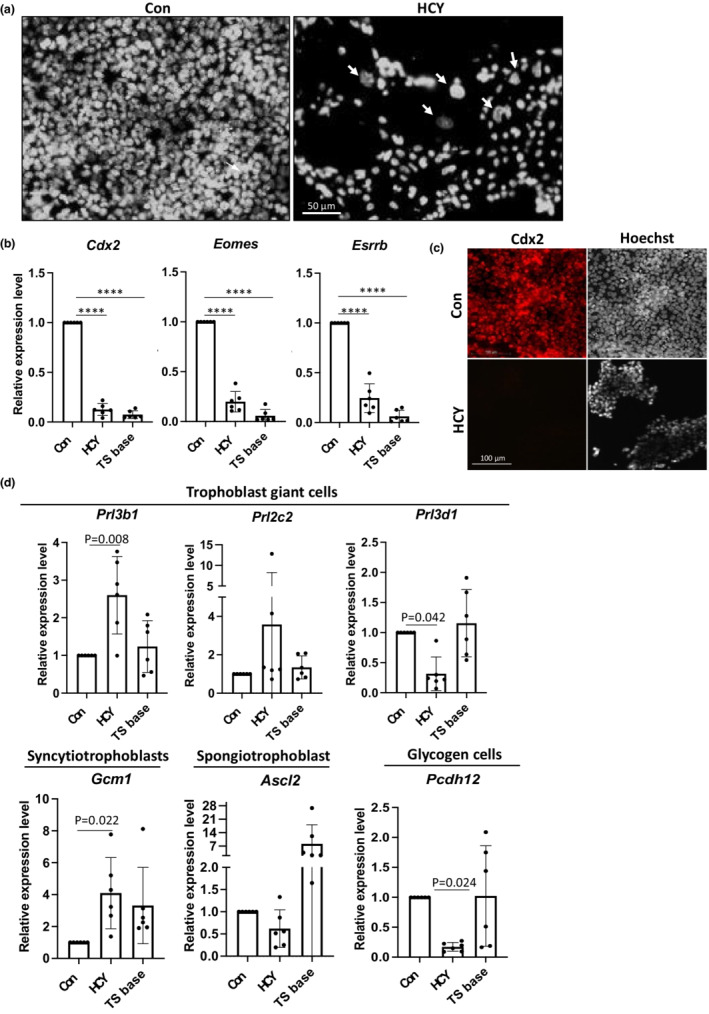
High HCY reduces trophoblast stem cells stemness while promotes trophoblast differentiation. TSCs were treated either in the presence or absence of 10 mM HCY in complete medium or in TS base medium for 48 h. (a, c) Cells were fixed for 4 immunostaining of Cdx2 and counterstained with Hoechst 33342 for nuclei. All images were taken using same settings with a Zeiss fluorescent microscope. (a) HCY‐treated TSCs exhibit some cells with “giant” nuclei. Scale bar = 50 μm. (b) Gene expression analysis was via RT‐qPCR of trophoblast stem cell markers including Cdx2, Eomes and Esrrb. (c) Loss of Cdx2 protein in TSCs after HCY treatment. Scale bar = 100 μm. (d) Gene expression analysis via RT‐qPCR of trophoblast giant cell (TGC) markers including *Prl3b1*, *Prl3d1* and *Prl2c2*, spongiotrophoblast marker *Ascl2*, glycogen trophoblast cell marker *Pcdh12*, and labyrinth progenitor cells cell marker *Gcm1*. For (b) and (d), data are presented as relative expression level to untreated control (Con) (mean ± SD) *n* = 6 biological replicates per group. RM One‐way ANOVA with Dunnett's multiple comparisons test was performed in comparison to Con except *Prl3d1*, *Prl2c2* and *Ascl2*, in which non‐parametric Friedman test with Dunnett's multiple comparisons test was performed. ****p < 0.0001.

We next assessed whether HCY induces differentiation of TSCs into trophoblast sublineages after 48 h incubation. First, we observed cells exhibiting an enlarged nucleus, a hallmark of differentiated TGCs (Figure 4a, arrows). To examine whether the stem cell population was maintained in TSCs cultured in the presence of HCY, known stem cell markers were assessed using RT‐qPCR. Treatment with HCY induced an approximately 0.8‐fold reduction (*p* < 0.0001) in mRNA expression of *Cdx2*, *Esrrb* and *Eomes* compared to non‐treated controls (Con) (Figure 4b). These decreases in expression were comparable to TSCs cultured without growth factors (Figure 4b, TS base), which is known to induce TSC differentiation (Tanaka et al., 1998). Immunostaining confirmed the loss of Cdx2 protein in the HCY‐treated TSCs in complete medium (Figure 4c).

We next examined changes of expression of six trophoblast‐specific lineage markers, including TGCs markers: *Prl3d1* (also known as PI1), *Prl3b1* (also known as Pl2) *and Prl2c2* (also known as Plf); labyrinth progenitor cell marker: *Gcm1*; spongiotrophoblast marker: *Ascl2*; and glycogen cell marker: *Pcdh12*. After 48 h, HCY induced differential expression of different TGC markers; increased expression of *Prl3b1* by 2.9 fold (*p* = 0.008) and a trend to increase of *Prl2c2* by 3.6 fold (*p* = 0.166) while decreased expression of *Prl3d1* by 0.7 fold (*p* = 0.042) (Figure 4d). Additionally, HCY elevated expression of *Gcm1* by 4.1 fold (*p* = 0.022) while reducing *Pcdh12* expression by 0.83 fold (*p* = 0.024) (Figure 4d). It had no effect in expression of *Ascl2* (Figure 4d). The contrasting expression profile of the trophoblast cell lineage markers following HCY treatment and growth factor withdrawal indicated these stimuli triggered different mechanisms modulating TSC differentiation. Differentiation of trophoblast requires a considerable period of time (Erlebacher et al., 2004; Tanaka et al., 1998). Our 48 h treatment is a relatively short period for studying differentiation of trophoblast and this might account for the low magnitude of the changes observed.

To confirm whether high concentrations of HCY promote precocious differentiation of TSCs in an ER stress‐dependent manner, TSCs were co‐cultured in HCY and chemical inhibitor of the UPR^ER^ signaling pathways. TUDCA, which effectively suppressed ER stress in the HCY‐treated blastocysts, failed to do so in TSCs, as indicated by the increase in *Grp78* expression in TSCs co‐treated with HCY and TUDCA for 48 h compared to non‐treated controls (Figure S4). Although there are inhibitors specific for different arms of UPR^ER^ pathways such as 4μ8C (Ire1α inhibitor), and sephin1 (holophosphatase inhibitor in Perk pathway), they are not suitable for this study. 4μ8C inhibits the Ire1α pathway while greatly activating the Perk pathway (Capatina et al., 2021), while sephin1 has a short half‐life (Capatina et al., 2021; Das et al., 2015). Nevertheless, we have demonstrated that sephin1 abolishes tunicamycin‐activated UPR^ER^ signaling pathways in TSCs (Capatina et al., 2021). Despite being aware that differentiation into the majority of trophoblast sublineages may not occur during the short experimental duration, we attempted using sephin1 to inhibit TSC differentiation. Co‐treatment of TSCs with HCY and sephin1 for 12 h, the maximal incubation time possible with Sephin1 (Das et al., 2015), did suppress the ER stress response and only increased expression of *sXbp1* and *Chop* (Figure S5a) was observed compared to non‐treated controls. The 12 h treatment of TSCs with HCY was sufficient to promote the expression of the TGC marker gene (*Prl3b1)* by 2‐fold (*p* = 0.051) (Figure S5b). Co‐treatment with HCY and sephin1 normalized *sXbp1* and *Chop* expression (Figure S5a), and crucially abolished the change in *Prl3b1* expression (*p* = 0.031; Figure S5b). These results provide support that the differentiation of TSCs promoted by HCY is likely mediated in a UPR^ER^‐dependent manner, but further studies are required when a universal inhibitor of the UPR^ER^ pathways becomes available.

## 4 DISCUSSION

This is the first study to elucidate the detrimental effects of hyperhomocysteinemia on development of the TE layer of blastocysts and differentiation of TSCs. The influence of HCY on these processes is mediated in part through UPR^ER^ signaling pathways following the induction of ER stress. Exposure of blastocysts to high concentrations of HCY ex vivo induces ER stress and is associated with a reduction in cell number in the TE layer attributed in part to apoptosis and the promotion of trophoblast differentiation. These phenotypes are partially reversed by alleviation of ER stress using the chemical chaperone TUDCA. The influence of ER stress on the development of blastocysts and trophoblast differentiation has been demonstrated in our previous study in TSCs with ER stress inducers and in the *Eif2s1* ^tm1RjK^ transgenic model of chronic ER stress (Capatina et al., 2021). Importantly, TUDCA alleviates ER stress in HCY‐treated blastocysts and reduces cell death and inhibits ER stress in the *Eif2s1* ^tm1RjK^ homozygous placenta, leading to improved pregnancy outcomes. Ursodeoxycholic acid (UDCA), a derivative of TUDCA, has already been used for treatment of intrahepatic cholestasis during human pregnancy (Hague et al., 2021). Our results may therefore provide new insights for potential therapeutic interventions to improve pregnancy outcomes in women with hyperhomocysteinemia.

The precise mechanisms by which HCY modulates TSC proliferation or self‐renewal and trophoblast lineage differentiation remain to be elucidated. Protein misfolding caused by homocysteinylation provides a potential direct stimulus to provoke ER stress. Alternatively, there may be indirect mechanisms, such as through disruption of intracellular Ca^2+^ homeostasis or reduction–oxidation (redox) balance which may perturb ER homeostasis and result in protein misfolding (Feige & Hendershot, 2011; Krebs et al., 2015; Malhotra et al., 2008). Treatment with HCY upregulates intracellular ROS and Ca^2+^ ions in cells in a dose‐dependent manner (Moshal et al., 2006) by promoting calcium release from its intracellular storage compartments (Mujumdar et al., 2000). We have previously reported that oxidative stress is a strong inducer of ER stress in trophoblastic cells (Yung et al., 2007, 2008), and that trophoblastic cell apoptosis and proliferation are regulated in an ER stress‐severity dependent manner (Yung et al., 2008). Loss of Ca^2+^ homeostasis may also activate other signaling pathways, for it is notable that influx of Ca^2+^ through the Erb B receptor is crucial for heparin‐binding EGF‐like growth factor‐induced trophoblast differentiation (Wang et al., 2000).

ER stress associated with HCY‐treatment of blastocysts and *Eif2s1* ^tm1RjK^ homozygous blastocysts resulted in similar effects on the TE in blastocysts (Capatina et al., 2021). However, alleviation of ER stress by TUDCA was only sufficient to partially restore trophoblast populations in the *Eif2s1* ^tm1RjK^ homozygous mutant placentas at mid‐gestation and did not increase cell numbers in the TE layer in HCY‐treated blastocysts (Capatina et al., 2021). These findings suggest the presence of ER stress‐independent pathways in the regulation of cell proliferation in response to HCY. We hypothesize an alternative mechanism of hyperhomocysteinemia involving a direct effect of protein homocysteinylation on growth factor receptors, leading to loss of function and attenuation of intracellular signals involved in cell growth and proliferation.

Once activated, the UPR^ER^ pathways may influence the transcriptional network regulating TSC maintenance and differentiation. The promoter of the *Pl1* (*Prl3d1*) gene is a putative target of Atf4 (Rouillard et al., 2016). Interestingly, although Atf4 is increased in tunicamycin‐induced ER stress associated with increased *Prl3d1* expression (Capatina et al., 2021), our results showed the opposite effect in HCY‐treated TSCs, indicating that additional factors are likely modulating the role of Atf4 in regulation of *Prl3d1* gene expression. Furthermore, ER stress can also directly induce *Gcm1* expression in trophoblast cells (Schubert et al., 2008), mediated through Oasis, a potential UPR sensor (Kondo et al., 2005). Oasis belongs to the Creb/Atf family of transcriptional factors (Saito et al., 2012). Indeed, we found increased *Gcm1* expression with high *Atf4* following HCY treatment.

The implications of our findings that ER stress modulates blastocyst development and trophoblast differentiation are not limited to hyperhomocysteinemia. The UPR pathways are a point of convergence of a variety of physiological and environmental stresses that act collectively through the integrated stress response (ISR) (Ron & Walter, 2007). Hence, the concept could also be potentially applied to poor pregnancy outcomes associated with other pre‐gestational health conditions such as obesity, diabetes, and viral infections, as well as pregnancy in unfavorable environments, including famine and at high altitude, and artificial reproductive technologies. The ISR induces phosphorylation of eukaryotic initiation factor subunit alpha (eIF2α), and activation of the Atf4 axis, through four upstream kinases; HRI (eIF2α kinase heme‐regulated inhibitor), GCN2 (general control nonderepressible 2), PKR (double‐stranded RNA [dsRNA]‐dependent protein kinase) and PERK, in response to hypoxia, nutrient deprivation, viral infection, and oxidative stress, respectively (Wek et al., 2006). Indeed, severe hypoxia, nutrient deprivation, and osmotic stress all lead to a loss of stemness and premature differentiation of the trophoblast lineage (Rappolee et al., 2013; Yang et al., 2017). Similar to the findings presented here, exposure to hyperosmotic stress, which activates ER stress through HRI (Pakos‐Zebrucka et al., 2016), down‐regulated *Cdx2* transcripts and caused the up‐regulation of genes, such as *Prl3d1* & *Prlc2c*, associated with differentiation into TGCs (Liu et al., 2009).

A weakness of our study is the inability to directly investigate whether alleviation of ER stress in animals with hyperhomocysteinemia improves pregnancy outcome. We found that current animal models of hyperhomocysteinemia, such as the *Mtrr* ^*−/−*^ and *CBS* ^*−/−*^ transgenics or dietary manipulations (e.g. folate/vitamin B12 deficiency or high‐methionine diet) are closely associated with adverse genetic/epigenetic influences on embryonic development and vascular pathologies (Beard & Bearden, 2011; Padmanabhan et al., 2013; Troen et al., 2003). They were therefore not suitable for our study. A new animal model free from these confounders will need to be introduced in the future. Lack of universal inhibitor for UPR^ER^ pathways also prevented us to further explore how HCY modulates differentiation of trophoblast lineage in TSCs.

To conclude, our results demonstrate that exposure to high concentrations of HCY during the earliest stages of pregnancy affects trophoblast differentiation and has detrimental consequences for pregnancy outcome. Stress can activate many intracellular signaling responses (Rappolee et al., 2013), but our results indicate that the effects are partially mediated through ER stress response pathways. The finding that TUDCA can block many of the changes offers the prospect of novel therapeutic interventions. TUDCA is used as a food supplement. Administration of TUDCA during pregnancy did not affect litter size and reduces resorption rates, limiting safety concerns (Capatina et al., 2021). It is notable that TUDCA improves blastocyst development rate, the proportion of TE cells, and cell survival in bovine embryos cultured in vitro (Yoon et al., 2014). Therefore, TUDCA administration to pregnant women may improve adverse pregnancy outcomes arising from hyperhomocysteinemia.

## AUTHOR CONTRIBUTIONS

NC performed experiments, analyzed and interpreted the data, and contributed in manuscript preparation. GJB provided valuable advice and critically reviewed the manuscript. HWY conceived the study, designed, directed, and performed experiments, analyzed and interpreted the data, and wrote the manuscript. All authors edited the manuscript and approved the final version.

## FUNDING INFORMATION

The study was funded by Centre for Trophoblast Research including a PhD studentship for NC. NC also received a Lucy Cavendish College award.

## CONFLICT OF INTEREST

The authors declare that no conflicts of interest exist.

## Supporting information


Appendix S1

## Data Availability

The data that support the findings of this study are available in the published figures.
