# Supplementary material for: Elevated homocysteine activates unfolded protein responses and causes aberrant trophoblast differentiation and mouse blastocyst development
Source: Physiol Rep. 2022 Sep 18;10(18):e15467. doi: 10.14814/phy2.15467 (PMC9483615; doi:10.14814/phy2.15467)
Supplement: Supplementary file 1 — Appendix S1 [file PHY2-10-e15467-s001.pdf]

Suppl. Figure S1

A

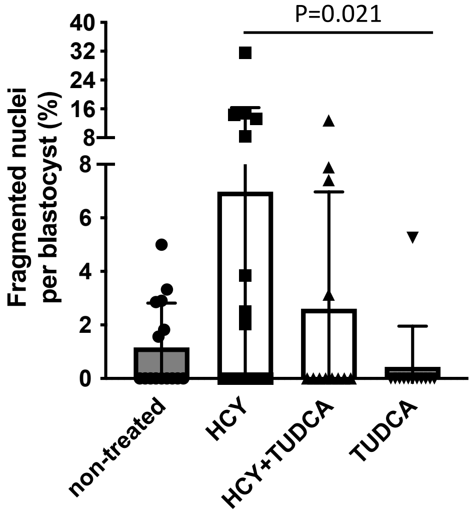

B

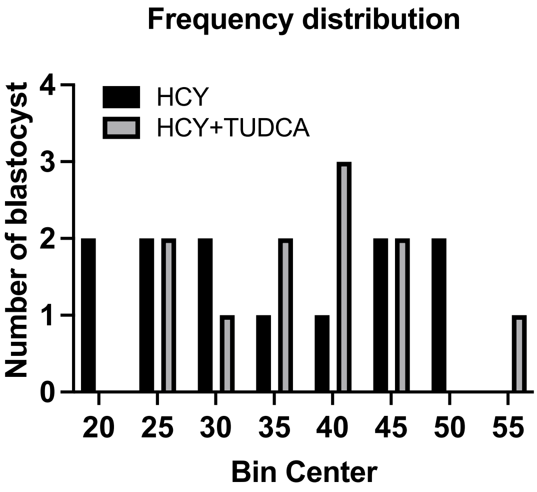

Suppl. Fig. S1: TUDCA partially abolishes HCY induced apoptotic death cells in blastocysts as well as slightly restores number of cells in blastocysts after HCY treatment. Morulas were harvested from C57/Bl6 females at 2.5 dpc and were cultured in KSOM medium in the presence or absence of HCY (100 µM) or TUDCA (500 µM) or a combination of both for 48 h. **A)**TUDCA reduces number of cells with fragmented nuclei in HCY-treated blastocysts. Blastocysts were fixed and stained with Hoechst 33342. Number of fragmented nuclei and a total number of nuclei in each balstocyst were counted separately. Data is expressed as mean±SD. Kruskal-Wallis test with Dunn'smultple comparison test was performed. **B)**TUDCA reduces number of HCY-treated blastocysts fewer than 30 cells. Frequency distribution graph was plotted with bin center (number of cells in blastocyst) as 5.

Suppl. Figure S2

A

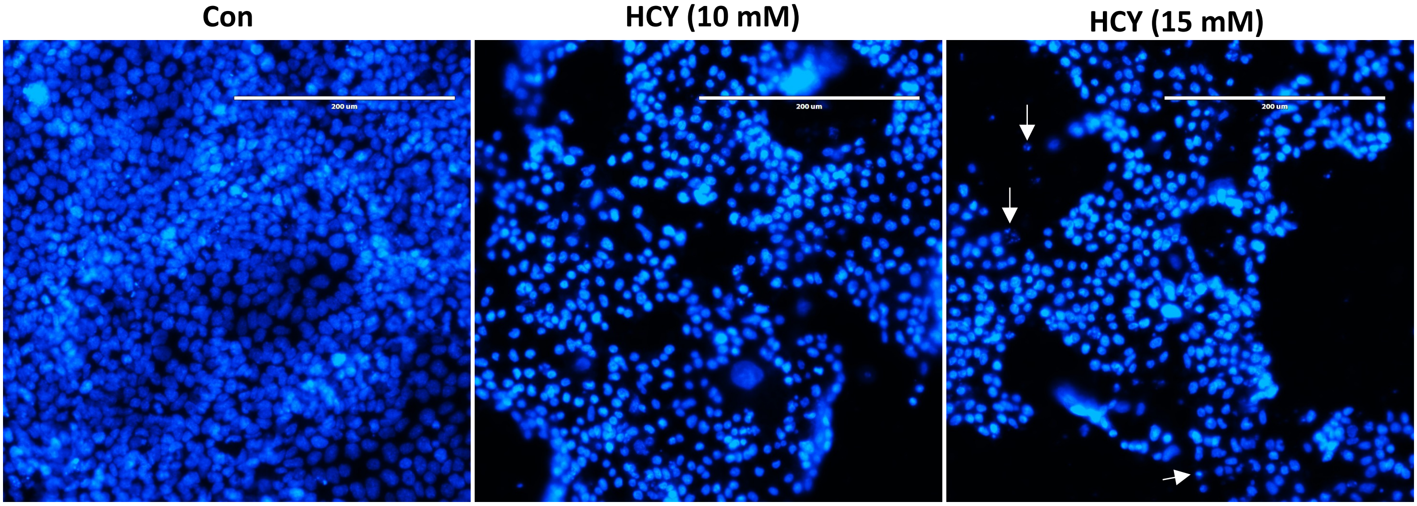

B

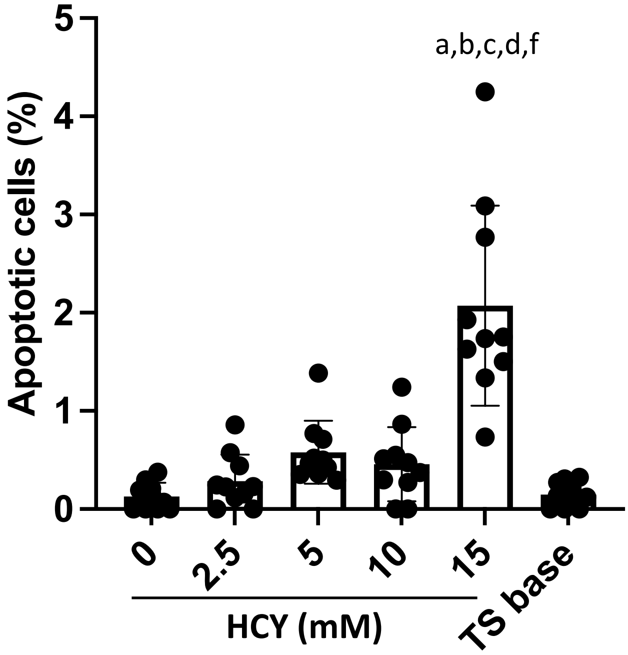

Suppl. Fig. S2: High Homocsyteine treatments have little impact in cell death of mouse trophoblast stem cells. TSCs were treated with an increase concentration of HCY from 2.5 to 15 mM for 48 h. **A)** Hoechst 33342 staining was performed and number of fragmented or highly condensed nuclei (arrows) and a total number of nuclei were counted. **B)** Percentage of apoptotic cells was calculated from the ratio between number of fragmented or condensed nuclei and the total number of nuclei and is expressed as mean±SD, n= 10 fields from two independent experiments. Ordinary one way ANOVA with Tukey's mulitple comparison test to untreated control (0). “a”, “b”, “c”, “d” and “e” indicates P<0.05 compared to 0, 2.5, 5, 10 and 15 mM respectively. .

Suppl. Figure S3

A

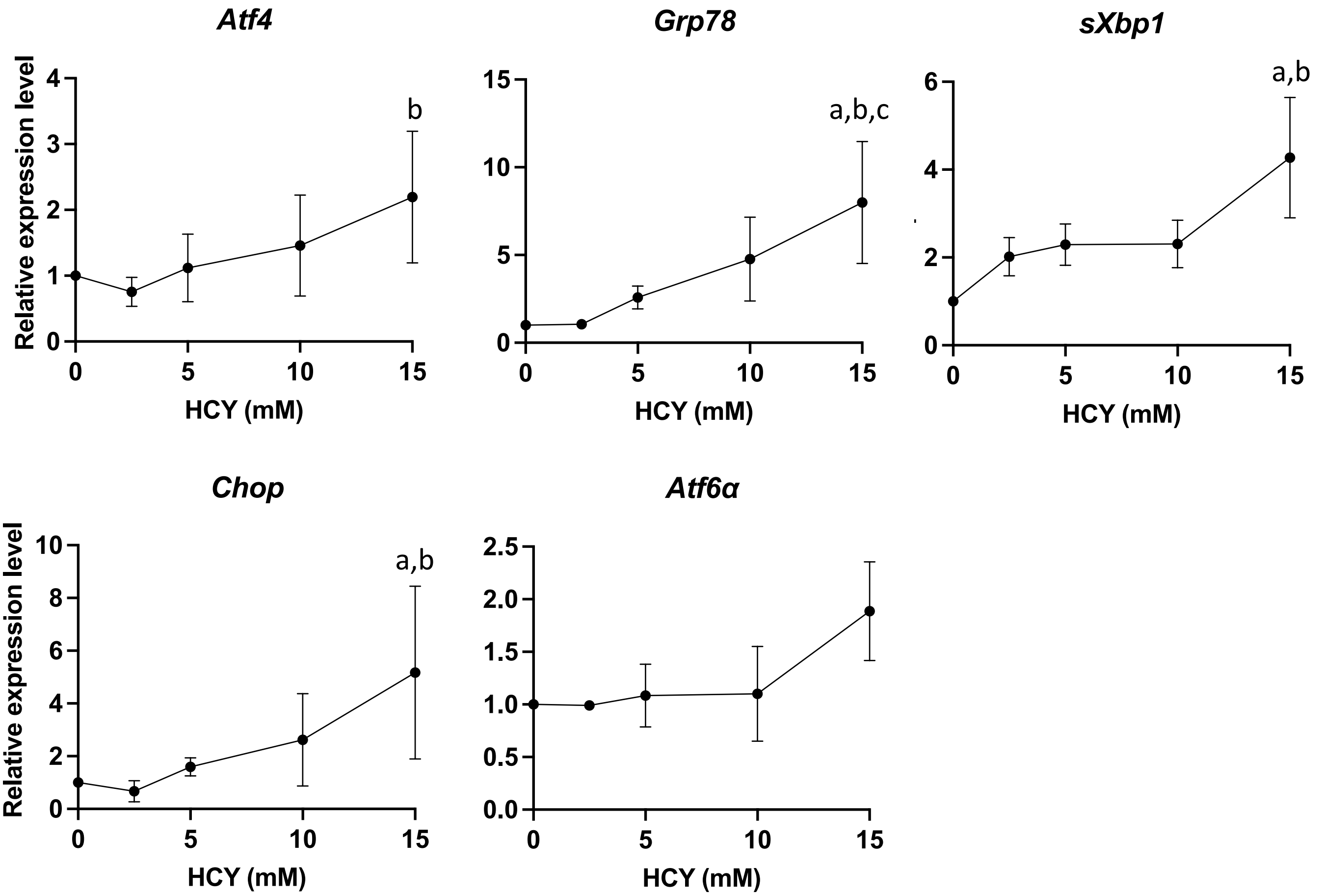

B

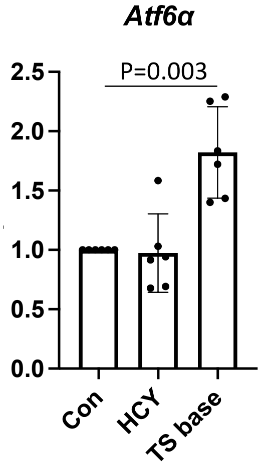

Suppl. Fig. S3: HCY induces UPR^{ER} signalling pathways in a dose-dependent manner.TSCs were treated with an increase concentration of HCY from 2.5 to 15 mM for 48 h. **A)** Total RNA was isolated after 48 h for RT-qPCR analysis of ER stress markers' expression. Data are presented as relative ratio to untreated control (Con) (mean±SD), n=3 independent experiments. RM one-way ANOVA with Tukey's multiple comparisons test was performed in comparison to Con. "a", "b", "c", "d" and "e" indicates P<0.05 compared to 0, 2.5, 5, 10 and 15 mM respectively. **B)** *Atf6α* transcript is significant increased in TS base treated TSCs despite no significant change in all treated concentration of HCY after 48 h. Data is expressed as mean±SD, n=6. RM One-way ANOVA with Tukey's multiple comparisons test was performed in comparison to Con.

Suppl. Figure S4

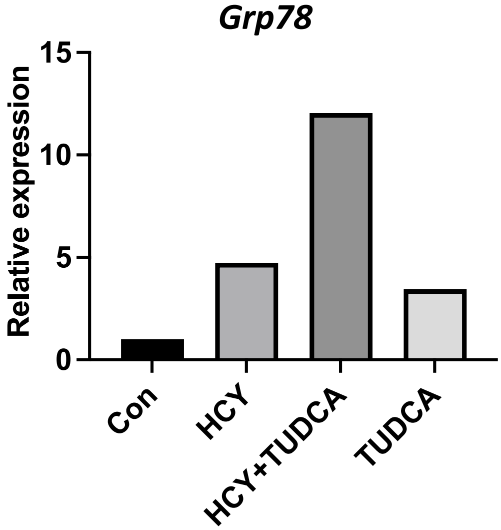

Suppl. Fig. S4. TUDCA fails to alleviate HCY-mediated ER stress in TSCs. TSCs were pre-treated with TUDCA for 1 h, followed by HCY 10 mM treatment with or without the TUDCA for 12 h. Total RNA was collected and gene expression of *Grp78* transcript was analysed. Data is presented as relative ratio to non-treated control (Con) transcript levels, which was normalized to 1, n=1.

Suppl. Figure S5

A

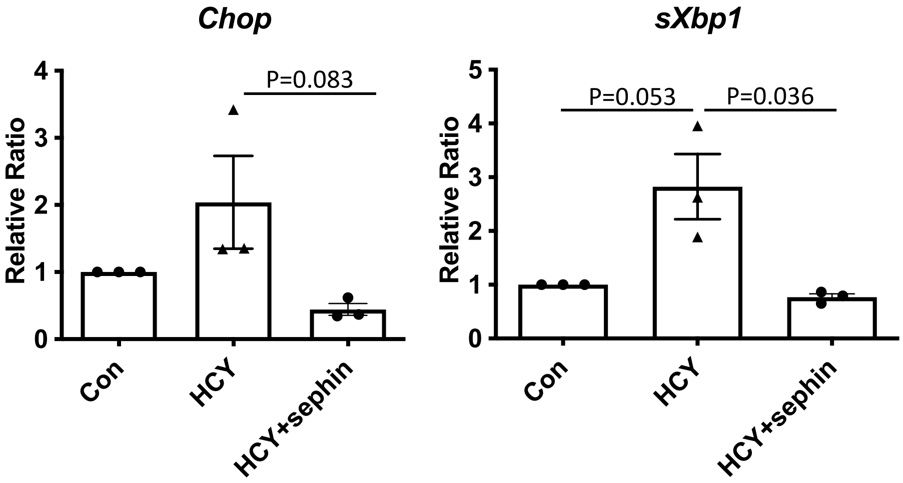

B

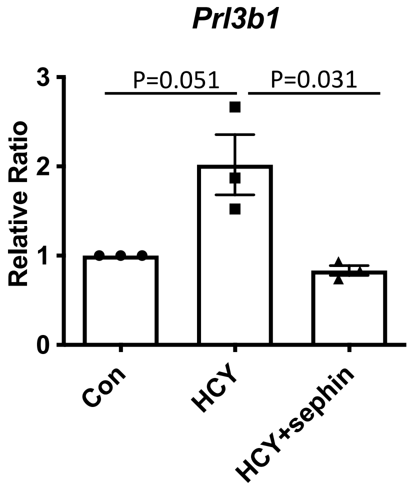

Suppl. Fig. S5. HCY regulates mouse trophoblast stem cell differentiation *in vitro* through ER stress response pathway. TSCs were pre-treated with Sephin1, an ER stress inhibitor for 1 h, followed by HCY 10 mM treatment with or without the inhibitor for 12 h. Total RNA was collected and gene expression analysis was performed via RT-qPCR. Specific genes assessed were **A)** ER stress marker, *Chop and sXbp1*; and **B)** Trophoblast giant cell marker, *Prl3b1*. All data are presented as relative ratio to DMSO vehicle control (Con) transcript levels, which was normalized to 1 (mean±SD), n=3 biological replicates. RM one-way ANOVA with Tukey's multiple comparison test.

Table 1. Primer pair sequences, annealing temperatures, and the amplicon sizes used.

Primer name	Primer sequence	Manufacturer/ Reference	Ann t (^{O}C)	Amplicon size (bp)
Ascl2 For	5'-AGCCCGATGGAGCAGGAG-3'	(Branco *et al.*, 2016)	64	197
Ascl2 Rev	5'-CCGAGCAGAGGTCAGTCAGC-3'			
Atf4 For	5'-GAGCTTCCTGAACAGCGAAGTG-3'	(Yu *et al.*, 2013)	64	112
Atf4 Rev	5'-TGGCCACCTCCAGATAGTCATC-3'			
Cdx2 For	Mm01212280_m1	ThermoFisher, 4331182	60	64
Cdx2 Rev				
Chop For	5'-CTGCCTTTCACCTTGGAGAC-3'	(Rutkowski *et al.*, 2006)	58	118
Chop Rev	5'-CGTTTCCTGGGGATGAGATA-3'			
Eomes For	Mm01351985_m1	ThermoFisher, 4331182	60	58
Eomes Rev				
Esrrb For	5'-AGTACAAGCGACGGCTGG-3'	(Latos *et al.*, 2015)	58	103
Esrrb Rev	5'-CCTAGTAGATTCGAGACGATCTTAGTCA-3'			
Gcm1 For	5'-CATCTACAGCTCGGACGACA-3'	(He *et al.*, 2008)	58	144
Gcm1 Rev	5'-CCTTCCTCTGTGGAGCAGTC-3'			
Grp78 For	5'-CTGAGGCGTATTTGGGAAAG-3'	(Chang *et al.*, 2012)	58	120
Grp78 Rev	5'-TCATGACATTCAGTCCAGCAA-3'			
Hprt For	Mm01545399_m1	ThermoFisher, 4331182	60	81
Hprt Rev				
Pcdh12 For	5'-GAAGAGCTGTCGAGCCTGTT-3'	(Branco *et al.*, 2016)	64	101
Pcdh12 Rev	5'-GTGAGGGGCAATGACAATCT-3'			
Perk For	5'-CTTAATCCATTCTCCTTCTAGG-3'	Designed by Sigma	58	156
Perk Rev	5'-TAGTATGGCAGATAGTAACCG-3'			
Prl3d1 For	5'-TTATCTTGGCCGCAGATGTGT-3'	(Murray *et al.*, 2016)	58	109
Prl3d1 Rev	5'-GGAGTATGGATGGAAGCAGTATGAC-3'			
Prl3b1 For	5'-GCACTCGGGGAACAGCAGCC-3'	(Murray *et al.*, 2016)	64	117
Prl3b1 Rev	5'-ACTGCCAGCAACAGGAGTGCC-3'			
Prl2c2 For	5'- AACGCAGTCCGGAACGGGG-3'	(Murray *et al.*, 2016)	58	148
Prl2c2 Rev	5'- TGTCTAGGCAGCTGATCATGCCA-3'			
Sdha For	5'-TGGTGAGAACAAGAAGGCATCA-3'	(Murray *et al.*, 2016)	58	95
Sdha Rev	5'-CGCCTACAACCACAGCATCA-3'			
sXbp1 For	5'-GAGTCCGCAGCAGGTG-3'	(Zhang & Kaufman, 2008)	58	65
sXbp1 Rev	5'-GTGTCAGAGTCCATGGGA-3'			

References:

Branco MR, King M, Perez-Garcia V, Bogutz AB, Caley M, Fineberg E, Lefebvre L, Cook SJ, Dean W, Hemberger M & Reik W. (2016). Maternal DNA Methylation Regulates Early Trophoblast Development. *Dev Cell* **36,** 152-163.

Chang JS, Ocvirk S, Berger E, Kisling S, Binder U, Skerra A, Lee AS & Haller D. (2012). Endoplasmic reticulum stress response promotes cytotoxic phenotype of CD8αβ+ intraepithelial lymphocytes in a mouse model for Crohn's disease-like ileitis. *J Immunol* **189,** 1510-1520.

He S, Pant D, Schiffmacher A, Meece A & Keefer CL. (2008). Lymphoid enhancer factor 1-mediated Wnt signaling promotes the initiation of trophoblast lineage differentiation in mouse embryonic stem cells. *Stem Cells* **26,** 842-849.

Latos PA, Goncalves A, Oxley D, Mohammed H, Turro E & Hemberger M. (2015). Fgf and Esrrb integrate epigenetic and transcriptional networks that regulate self-renewal of trophoblast stem cells. *Nat Commun* **6,** 7776.

Murray A, Sienerth AR & Hemberger M. (2016). Plet1 is an epigenetically regulated cell surface protein that provides essential cues to direct trophoblast stem cell differentiation. *Sci Rep* **6,** 25112.

Rutkowski DT, Arnold SM, Miller CN, Wu J, Li J, Gunnison KM, Mori K, Sadighi Akha AA, Raden D & Kaufman RJ. (2006). Adaptation to ER stress is mediated by differential stabilities of pro-survival and pro-apoptotic mRNAs and proteins. *PLoS Biol* **4,** e374.

Yu S, Zhu K, Lai Y, Zhao Z, Fan J, Im HJ, Chen D & Xiao G. (2013). atf4 promotes β-catenin expression and osteoblastic differentiation of bone marrow mesenchymal stem cells. *Int J Biol Sci* **9,** 256-266.

Zhang K & Kaufman RJ. (2008). Identification and characterization of endoplasmic reticulum stress-induced apoptosis in vivo. *Methods Enzymol* **442,** 395-419.
